# Development of a foot and ankle strengthening program for the treatment of plantar heel pain: a Delphi consensus study

**DOI:** 10.1186/s13047-023-00668-2

**Published:** 2023-10-03

**Authors:** John W. A. Osborne, Hylton B. Menz, Glen A. Whittaker, Karl B. Landorf

**Affiliations:** 1 Discipline of Podiatry, School of Allied Health, Human Services and Sport, La Trobe University, Victoria, 3086 Australia; 2 La Trobe Sport and Exercise Medicine Research Centre, La Trobe University, Victoria, 3086 Australia

**Keywords:** Delphi study, Feet, Lower extremity, Muscle strength, Strength training, Plantar fasciitis, Plantar heel pain

## Abstract

**Background:**

People with plantar heel pain (PHP) have reduced foot and ankle muscle function, strength and size, which is frequently treated by muscle strengthening exercises. However, there has been little investigation of what exercises are used and there is no sound evidence base to guide practice. This study aimed to develop a consensus-driven progressive muscle strengthening program for PHP.

**Methods:**

Thirty-eight experts were invited to participate in the study over three rounds. Round 1 was an open-ended questionnaire that provided the core characteristics of progressive strengthening programs designed for three different adult patient types with PHP (younger athletic, overweight middle-aged, older), which were presented as vignettes. In Round 2, experts indicated their agreement to the proposed exercises and training variables. In Round 3, experts were presented with amendments to the exercises based on responses from Round 2 and indicated their agreement to those changes. Consensus was achieved when > 70% of experts agreed.

**Results:**

Two experts were ineligible and 12 declined, leaving 24 (67%) who participated in Round 1. Eighteen (75%) completed all three rounds. From Round 1, progressive strengthening programs were developed for the three vignettes, which included 10 different exercises and three training variables (sets / repetitions, weight, and frequency). In Round 2, 68% (*n* = 17) of exercises and 96% (*n* = 72) of training variables reached consensus. In Round 3, only exercise changes were presented and 100% of exercises reached consensus.

**Conclusions:**

This study provides three progressive strengthening programs agreed to by experts that can be used in future clinical trials to determine the effectiveness of muscle strengthening for PHP. In addition, clinicians could use the programs as part of a rehabilitation strategy with the caveat that they may change as more research is conducted.

**Supplementary Information:**

The online version contains supplementary material available at 10.1186/s13047-023-00668-2.

## Background

Plantar heel pain (PHP) is a common condition affecting the lower limb. It is most prevalent amongst middle aged adults affecting between 4 and 7% of the general population as well as accounting for up to 8% of running related injuries [1–3]. Importantly, PHP can have detrimental impacts including pain, reduced function and negative psychological effects [1, 4, 5].

Typically, those most affected with PHP are more likely to be obese and stand for longer periods of time [6, 7]. In addition, athletically active individuals (e.g. runners) are also commonly affected [3, 8, 9]. However, recent research has also found that reduced muscle function, strength and size is associated with people with PHP compared to people without [10]. Commonly used interventions [11–14] do not seem to address this association and they only have limited effectiveness with 45% of cases reporting they still have symptoms 10 years after the initial onset of symptoms [15]. It is possible, therefore, that addressing the associated reduced muscle function and strength may improve treatment outcomes.

Improving muscle strength has been used successfully for managing other lower limb complaints [16]. However, while existing strengthening programs for PHP are frequently prescribed, these programs are inconsistent and have undergone limited rigorous evaluation of their effectiveness [17–20]. These limitations include: no consensus on which muscles to target, what exercises to use, and what program of repetitions, resistance, and frequency to implement. Accordingly, clinicians have no evidence-based guidelines to follow when recommending strengthening programs for PHP. Without such guidelines, clinicians can only base their recommendations on anecdote and what little literature is available.

Therefore, to begin the process of acquiring an evidence base and developing guidelines, experts in the field need to combine their expertise to develop a strengthening program for PHP that can be rigorously evaluated and refined if necessary. Expert development will provide the essential initial step to rigorously investigate such a strengthening program; that is, to initially define and then refine the muscles to target, the exercises to use, and the program of repetitions, resistance, and frequency to apply.

With this in mind, this study aimed to seek information from experts to develop a consensus-driven progressive strengthening program for PHP. The findings of this study may inform future clinical trials that evaluate the effectiveness of strength training for PHP.

## Methods

### Study design

A modified Delphi technique [21, 22] was used. To maximise clarity and transparency, we followed recommendations from Junger et al. [23] when conducting and reporting this study.

### Expert panel selection

Currently, there is no consensus on the definition of an expert or the criteria to select experts [24]. To include experts with appropriate knowledge, experience, and a range of views, we sought experts from three categories (clinicians, academics, and course facilitators). A heterogeneous group of experts from varying disciplines, countries and professions were selected [24]. The following definitions were used.

#### Clinicians

Had a minimum of five years of clinical experience, saw a minimum of five patients with PHP per month, and/or prescribed foot and ankle muscle strengthening programs to at least five patients per month.

#### Academics

Were podiatry, physiotherapy, and exercise science university academics who had published a minimum of five peer-reviewed journal articles or textbook chapters related to PHP and/or foot and ankle muscle function, strength, or size.

#### Course facilitators

Were individuals who had delivered strengthening courses that related to PHP (but were not necessarily specific to PHP). The courses must have included foot and ankle muscle strengthening exercises or rehabilitation, run for a minimum of three hours or had equivalent online content, and were provided to health professionals.

Experts were recruited in January 2021 in four ways. The authors: (i) collated known clinicians, academics and course facilitators, (ii) searched for authors of articles with *PHP* and *strengthening exercises* as the topic, (iii) searched the Internet for courses offered to allied health professionals that related to lower limb, foot and ankle strengthening, and (iv) invited experts to provide details of other potential participants who met the eligibility criteria. All identified experts were invited to participate in the study via email.

### Procedure

The study was conducted between January and May 2021. Up to three reminders were sent to experts who did not complete a survey round within one week. All participants completed online consent. Data were collected and managed using REDCap (Research Electronic Data Capture) [25, 26].

#### Survey rounds

**Fig. 1:**
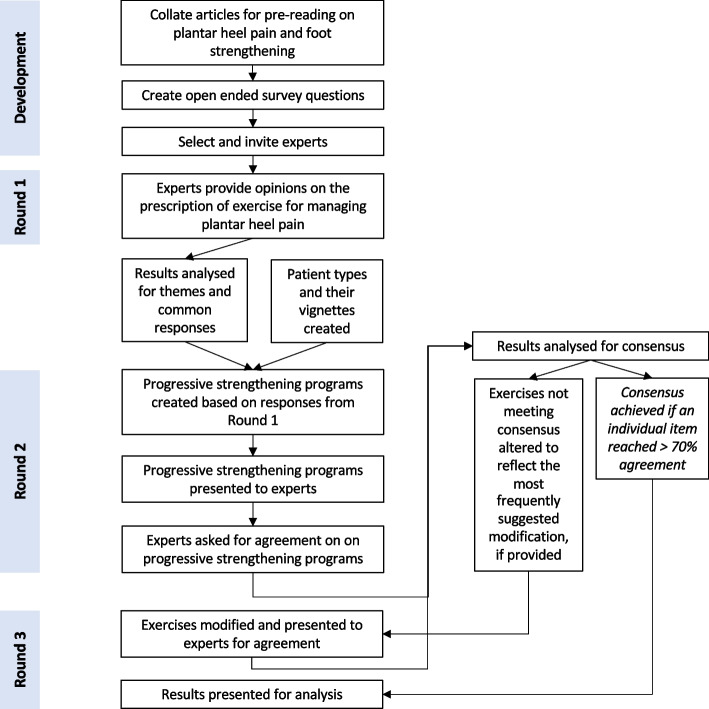
Flow chart illustrating progression through the study

The survey was conducted over three rounds (see Fig. 1 for flow of the study). Prior to each round, piloting was undertaken by staff within the Discipline of Podiatry at La Trobe University, who were not participants in the study. Prior to Round 1, participants were provided with access to relevant pre-reading to help inform their answers [10, 19].

*Round 1* was an open-ended questionnaire, which is considered appropriate to allow guidance for subsequent rounds of a survey [23]. Similar to other Delphi studies for musculoskeletal disorders [27, 28], a list of items (exercises and exercise variables) was developed based on participants’ answers from Round 1, which were then presented for agreement in Round 2. Where participants provided only a small number of exercises, which did not allow adequate progression or increase in difficulty from one stage of a strengthening program to the next, themes from Round 1 were used to provide guidance for extra exercise selection in Round 2.

For *Round 2*, experts were provided three different patient types presented as vignettes for which progressive strengthening programs were prescribed (Additional file 1). Exercises suggested for Round 2 were based on the most common answers from Round 1. Each vignette was based on a common clinical presentation of PHP and provided enough detail to develop a progressive strengthening program. The three vignettes were for a younger athletic adult, an overweight middle-aged adult, and an older adult. For each vignette, experts were asked to agree or disagree with the inclusion of each exercise and its training variables (sets and repetitions, weight, and frequency). They were also able to provide open-ended comments.

In *Round 3*, the same vignettes were used, but exercises that did not reach consensus in Round 2 were replaced with exercises that the experts suggested most frequently in that round. Experts were again asked to agree or disagree with the exercises presented to them. No exercise training variables were presented for consensus in Round 3.

#### Data handling and analysis

Statistical analysis was performed using Microsoft Excel (Microsoft Corporation, Redmond, Washington USA).

Quantitative and qualitative data from Round 1 were input to a Microsoft Excel spreadsheet under the headings of each question. Thematic analysis was performed on open-ended responses [29]. The results provided information that was used to modify the progressive strengthening programs associated with each vignette, including the exercise and program progression strategies. For dichotomous or single answer questions an accrued tally was performed (i.e., how many participants agreed). Where exercises were given different names but achieved the same movement (e.g., short foot and metatarsal doming), tallies were combined. Where an exercise provided variations on a movement (e.g., concentric heel raise, eccentric heel raise and double leg heel raise), tallies were made for both variations on the movement (e.g., concentric) and the primary movement (e.g., heel raise). Exercises and exercise training variables (e.g., sets, repetitions, and volume) used for inclusion in the progressive strengthening program were taken from the answers that accrued the highest tally.

A range of 50 to 95% has been used to define consensus in past Delphi studies [30]. Accordingly, we considered items that achieved > 70% agreement to have an acceptable level of agreement. Exercises that achieved < 70% agreement in Round 2 were altered to reflect the most frequently suggested modification, if provided, and agreement was subsequently sought in Round 3 for the modified exercise.

## Results

### Expert panel characteristics

Thirty-eight experts from seven different countries were invited to participate (Table 1). Two (5%) academic experts did not meet selection criteria due to having published less than five journal articles on PHP or muscle strengthening exercises. Of the remaining 36 experts, 24 (67%) agreed to participate.

**Table 1:** Expert panel characteristics (*n* = 24)

Characteristic	Category	Total
Country	Australia	8
	United Kingdom	4
	Netherlands	1
	Denmark	4
	Brazil	1
	United States of America	5
	Canada	1
Primary occupation	Podiatrist	7
	Physiotherapist	10
	Chiropractor	2
	Orthopaedic surgeon	2
	Sports/Exercise scientist	2
	Doctor of Medicine	1
Category of expert	Clinician	10
	Academic	13
	Course facilitator	1
Highest level of education	Bachelor	1
	Doctor of Chiropractic	2
	Doctor of Podiatric Medicine	1
	Masters	5
	PhD	15

### Round 1

All 24 participants who agreed to participate completed the open-ended questionnaire.

#### Exercise prescription

Twenty-two out of 24 (92%) respondents stated they would prescribe a progressive foot strengthening program for PHP. Of the two respondents who indicated they would not prescribe such a program, they agreed they would use a reloading strategy in the right circumstances:

‘…reloading with graded loading programme in standing (so arguably with a strength component in the exercise due to the functional nature of the exercise)…’.

and.

‘…I often prescribe whole body, or lower limb exercise or CV [cardio-vascular] exercise if the patient is coping with the core intervention components…’.

#### Strength training goals

Seven themes were extracted regarding the goals of strength training exercises for PHP including: addressing muscle weakness (*n* = 7), increasing tissue load/capacity (*n* = 6), reducing strain to the plantar fascia (*n* = 4), improving impact absorption of the foot (*n* = 3), improving function (*n* = 2), reducing arch deformation (*n* = 2), and reducing pronation (*n* = 1).

#### Indications and contraindications

The two most common responses regarding indications for a progressive strengthening program were that it can be applied to all patients (*n* = 6) and to athletic or physically active individuals (*n* = 4) (Additional file 2).

Only three contraindications for a progressive strengthening program were raised. Contraindications included the presence of a neurological (*n* = 1), bone (*n* = 1) or fat pad (*n* = 1) pathology (Additional file 2).

#### Exercise selection

The most common exercises were heel raise variations (*n* = 10), digital plantarflexion (*n* = 8), and the short foot exercise (*n* = 8) (Additional file 3).

#### Muscles to be targeted

The most common muscles to be targeted were foot intrinsics as a group (*n* = 6) (Additional file 4). Specific muscles mentioned included: calf (*n* = 2), flexor hallucis longus (*n* = 2), flexor digitorum brevis (*n* = 1), flexor digitorum longus (*n* = 1), tibialis posterior (*n* = 1), and adductors (*n* = 1) (Additional file 4). The term ‘adductors’ was mentioned but not defined as hip or foot adductors.

#### Movement concepts

Three themes emerged as movement concepts rather than specific exercises to be prescribed, including applying a talar neutral position (*n* = 3), foot core (*n* = 1), and toe posture (*n* = 1).

#### Dosage variables

The recommendations of variables to be used when prescribing a progressive strengthening program were sets and repetitions, time under tension, or using a repetition maximum. The most common dosage variable used was sets and repetitions (*n* = 14), followed by achieving a repetition maximum (*n* = 3), and time under tension (*n* = 2).

The most common number of sets was three (*n* = 5), four (*n* = 3), and then five (*n* = 3). The most common number of repetitions was 10, 12 and 15 (*n* = 5), however there was a range of repetitions offered (from 1 to 25). Occasionally, the repetition or set range was dependent on the exercise choice.

#### Progression of exercise

The most common responses for progressing difficulty of exercise were to increase volume (*n* = 8), weight (*n* = 5), and complexity (*n* = 3).

### Round 2

A three-stage progressive strengthening program was derived from the results of Round 1 for each vignette. Eighteen of the 24 (75%) experts completed Round 2 of the study, although one completed only the first vignette. Results for Round 2 are detailed below and in Table 2.

**Table 2:** Progressive strengthening programs with exercises, exercise variables and their levels of agreement from Round 2

Vignette (patient)	Stage	Exercise	%	Sets	Repetitions	%	Weight	%	Frequency	%
1. Younger athletic adult	Stage 1	Hallux plantarflexion banded	89%	4	6 to 12	100%	8 RM	81%	Daily	75%
		Digital plantarflexion banded	89%	4	6 to 12	100%	8 RM	81%	Daily	81%
		Heel raise seated digits dorsiflexed	67%	4	6 to 12	100%	8 RM	92%	Daily	67%
		Short foot exercise standing	67%	4	8 to 15	83%	BW	100%	Daily	92%
	Stage 2	Toe spread out	72%	3	8 to 15	85%	BW	100%	Daily	100%
		Heel raise standing digits dorsiflexed	78%	5	6 to 10	71%	8 RM	93%	Daily	86%
		Short foot exercise standing single leg	72%	3	8 to 15	85%	BW	92%	Daily	100%
	Stage 3	Heel raise standing single leg digits dorsiflexed	83%	5	8 to 15	87%	10 RM	80%	Daily	80%
		Walking lunges	83%	3	12 to 25	100%	20 RM	93%	Daily	80%
2. Overweight middle-aged adult	Stage 1	Towel scrunch with inversion and eversion	59%	4	8 to 12	100%	10 RM	80%	2nd Day	90%
		Hallux plantarflexion banded	83%	3	6 to 10	65%	8 RM	80%	2nd Day	73%
		Single leg standing unbalanced surface	53%	3	2 min	100%	BW	100%	2nd Day	78%
	Stage 2	Toe spread out	71%	3	6 to 10	100%	8 RM	83%	2nd Day	67%
		Heel raise standing digits dorsiflexed	71%	5	6 to 10	92%	8 RM	83%	2nd Day	75%
		Short foot exercise seated	59%	3	6 to 10	100%	8 RM	90%	2nd Day	80%
	Stage 3	Heel raise standing digits dorsiflexed	76%	5	6 to 10	92%	8 RM	85%	2nd Day	85%
		Walking lunges	88%	3	12 to 20	93%	15 RM	80%	2nd Day	87%
3. Older adult	Stage 1	Towel scrunches	53%	4	8 to 12	89%	10 RM	89%	2nd Day	89%
		Towel scrunch with inversion and eversion	41%	4	8 to 12	100%	10 RM	100%	2nd Day	100%
		Single leg standing	82%	2	2 min	79%	BW	100%	2nd Day	71%
	Stage 2	Toe spread out	71%	3	6 to 10	83%	8 RM	83%	2nd Day	67%
		Heel raise seated	76%	5	6 to 10	77%	8 RM	92%	2nd Day	77%
		Short foot exercise seated	65%	3	6 to 10	91%	8 RM	91%	2nd Day	82%
	Stage 3	Heel raise standing	82%	3	6 to 10	100%	8 RM	93%	2nd Day	79%
		Chair squat	82%	3	6 to 10	100%	8 RM	86%	2nd Day	93%

#### Younger athletic adult

Seven of 9 (78%) exercises achieved consensus. The exercises that did not achieve consensus were heel raise seated with digits dorsiflexed (67%) and short foot exercise while standing (67%). Twenty-six of 27 (96%) exercise training variables met consensus. The heel raise seated with digits dorsiflexed did not reach consensus for frequency of exercise (daily).

#### Overweight middle-aged adult

Five of 8 (63%) exercises achieved consensus. The exercises that did not achieve consensus were towel scrunch with inversion and eversion (59%), single leg standing on an unbalanced surface (53%), and short foot exercise seated (59%). Twenty-two of 24 (92%) exercise training variables reached consensus.

#### Older adult

Five of 8 (63%) exercises achieved consensus. The exercises that did not achieve consensus were towel scrunches (53%), towel scrunch with inversion and eversion (41%), and short foot exercise while seated (65%). All 24 (100%) exercise training variables reached consensus.

#### Progressions

The progressions of exercises and stages of the program had 54% agreement, so did not reach consensus in Round 2. The progressions were based on increasing repetitions (volume) first. This was outlined as: ‘*Each week the program progresses by adding two repetitions and keeping the weight and other variables the same. All participants begin on Stage 1 of the exercise regime. If there is no perceived difficulty or pain, then progression to Stage 2 and so on for Stage 3.*’

### Round 3

All 18 (100%) experts completed Round 3 of the study. The exercises that did not reach consensus in Round 2 were replaced in Round 3 with the exercises that were suggested most frequently by the experts in Round 2. For example, the towel scrunch with inversion and eversion did not meet consensus in Round 2 for the older adult (41%), so it was replaced with short foot exercise seated, which was the most frequently suggested replacement exercise. Following these replacements, all three progressive strengthening programs met consensus in Round 3 (Table 3).

**Table 3:** Selected exercises, stage of progression, and agreement from Round 3 (*n* = 18)

Vignette (patient)	Stage	Exercise	Agreement
1. Younger athletic adult	1	Heel raise	94%
		Short foot exercise seated	78%
2. Overweight middle-aged adult	1	Short foot exercise seated	83%
		Lesser digit plantarflexion banded	78%
	2	Short foot exercise standing	89%
3. Older adult	1	Digital plantarflexion banded	78%
		Short foot exercise seated	83%
	2	Digital plantarflexion banded	82%

#### Progressions

Exercise progressions were updated to increase weight and then functionality, rather than increase volume (repetitions) first. This change was made in response to feedback from the experts in Round 1. This progression strategy achieved 100% consensus. The final progressive strengthening programs are presented in Table 4.

**Table 4:** Strengthening programs that reached consensus after Round 3

Vignette (patient)	Stage	Exercise	Sets	Repetitions	Weight	Frequency
1. Younger athletic adult	Stage 1	Hallux plantarflexion banded	4	6 to 12	8 RM	Daily
		Digital plantarflexion banded	4	6 to 12	8 RM	Daily
		Heel raise	4	6 to 12	8 RM	Daily
		Short foot exercise seated	4	8 to 15	BW	Daily
	Stage 2	Toe spread out	3	8 to 15	BW	Daily
		Heel raise standing digits dorsiflexed	5	6 to 10	8 RM	Daily
		Short foot exercise standing single leg	3	8 to 15	BW	Daily
	Stage 3	Heel raise standing single leg digits dorsiflexed	5	8 to 15	10 RM	Daily
		Walking lunges	3	12 to 25	20 RM	Daily
2. Overweight middle-aged adult	Stage 1	Short foot exercise seated	4	8 to 12	10 RM	2nd daily
		Hallux plantarflexion banded	3	6 to 10	8 RM	2nd daily
		Lesser digit plantarflexion banded	3	2 min	BW	2nd daily
	Stage 2	Toe spread out	3	6 to 10	8 RM	2nd daily
		Heel raise standing digits dorsiflexed	5	6 to 10	8 RM	2nd daily
		Short foot exercise standing	3	6 to 10	8 RM	2nd daily
	Stage 3	Heel raise standing digits dorsiflexed	5	6 to 10	8 RM	2nd daily
		Walking lunges	3	12 to 20	15 RM	2nd daily
3. Older adult	Stage 1	Digital plantarflexion banded	4	8 to 12	10 RM	2nd daily
		Short foot exercise seated	4	8 to 12	10 RM	2nd daily
		Single leg standing	2	2 min	BW	2nd daily
	Stage 2	Toe spread out	3	6 to 10	8 RM	2nd daily
		Heel raise seated	5	6 to 10	8 RM	2nd daily
		Digital plantarflexion banded	3	6 to 10	8 RM	2nd daily
	Stage 3	Heel raise standing	3	6 to 10	8 RM	2nd daily
		Chair squat	3	6 to 10	8 RM	2nd daily

All participants begin on Stage 1 of the exercise regime. If there is no perceived difficulty or pain then progression to Stage 2 and so on for Stage 3

*RM *Repetition maximum, *BW *Body weight, 2nd daily = to perform every 2nd day

## Discussion

We conducted this Delphi study to gain consensus from a panel of experts on a program of strengthening exercises for PHP. Experts initially completed a questionnaire about important inclusions and exclusions in a progressive strengthening protocol. Before the next rounds, three vignettes were created to broadly represent three common but different patient types, recognising that one program may not be suitable across a range of sub-populations. Experts were then asked to agree or disagree, and provide feedback, for each of the proposed progressive strengthening programs. By the end of three rounds there was consensus on all three programs.

When experts were asked for their opinion on exercises (in Rounds 1 and 2) to be included three exercises were consistently recommended throughout the Delphi study, which were heel raises, digital plantarflexion and the short foot exercise. However, there was significant variation in how these exercises were described and applied. Heel raises, or exercises to improve calf strength, were the most commonly suggested exercise. However, a recent systematic review found that there is no difference in heel raise capacity between those with and those without PHP [10], so this recommendation may diverge from current evidence. Interestingly, both the heel raise exercise and the heel raise with the digits dorsiflexed exercise variation were occasionally not recommended by some experts due to perceived difficulty performing these exercises or provocation of symptoms. This inconsistency indicates that there is a need to better understand the role of exercises for PHP, including the barriers to using them. Furthermore, there is little robust evidence for the benefit of the exercise selections for those with PHP.

To ensure this program meets the optimum requirements of a strengthening program [31], we wanted to ensure that there was provision to progress the exercises and the programs with a focus on increasing strength. Muscle strength and electromyographic muscle activity can be increased by escalating exercise complexity towards more functional tasks and increasing the loads applied to the muscles during exercise [32, 33]. The results of this study recommend increases in weight (within each stage of the progressive strengthening program) and functionality (between individual stages of the program). The current American College of Sports Medicine (ACSM) Progression Models in Resistance Training for Healthy Adults Guidelines state that these loading principles should be applied to all strengthening programs if the goal is to increase strength [31]. We believe that the final programs in this Delphi study address techniques to progress patients with increased loads and functionality. However, further research evaluating their effectiveness would be beneficial.

In order to provide progression for increasing load on muscles, experts were given an opportunity to choose a repetition maximum to guide the weight choice for a given exercise. However, one concern from some of the experts in this study was the use of the expression ‘repetition maximum’, they stated this phrase was counterintuitive as it determines both the weight and the number of repetitions a patient could achieve for a given exercise. Upon reflection, we believe it is a limitation of the programs provided in our study and could cause some confusion. However, we wanted to quantify the amount of weight a patient could use to provide a method of progression in the program. A solution, for the clinician, may be to use an appropriate weight to meet the repetitions specified or use the repetition maximum in its place. The ACSM guidelines suggest a weight that is 60% of a one repetition maximum should be used to increase strength. However, a recent network meta-analysis suggests higher-load (> 80% of single repetition maximum) prescriptions maximise strength gains, and all prescriptions included in the analysis promote muscle hypertrophy [34].

Another method of progression, training frequency, was also debated within the expert panel. Some experts questioned whether daily or every second day was the most appropriate timeframe to facilitate increases in muscle strength with particular exercises. The ACSM guidelines do not specifically recommend how frequently exercises should be performed for maximum strength gains. The guidelines suggest up to four sets of an exercise per week for an untrained individual and up to 10 sets per week for trained individuals [31]. However, similar to the increasing load guidelines above, a recent network meta-analysis suggests that performing resistance training 2–3 times per week achieves the greatest muscle strength and hypertrophy gains [34].

Several strengths underpin this study. The experts selected were from a range of countries and were well distributed across professions dealing with PHP. In addition, the majority of experts had completed a PhD at the time of enrolment in the study, implying a deeper understanding of research and evidence. Finally, a relatively even distribution between clinical and academic experts in the study ensured a spread of knowledge between practical and theoretical approaches.

However, this study should also be viewed considering four limitations. Firstly, the overall response rate of those experts initially approached to participate was 67% and the retention rate across the three rounds was 75%. Delphi studies frequently have low response rates, however a retention rate of at least 70% or greater for each round is generally considered satisfactory [21, 22, 35]. It is possible that a larger sample may change the results of this study, although we believe our sample was broadly representative of the experts available. Secondly, we do not know the effectiveness of the exercise programs developed in this study. Future clinical trials that evaluate the effectiveness of these programs may change our understanding, which will subsequently influence future expert opinion. Nevertheless, as our knowledge currently stands, the programs we gained consensus for in this study provide a basis for further evaluation and offer clinicians a consensus-driven program to use for PHP. Thirdly, similar to many Delphi studies, we only considered items that achieved > 70% agreement to have an acceptable level of agreement, but we did not assess other factors such as percentage disagreement of, and variability in, answers from participants. Fourthly, this study only canvassed the opinions of experts, so we did not survey patients for their opinions regarding patient preferences. Indeed, one qualitative study found that participants with PHP reported that they ‘don’t feel as strong in (their) whole body’ and they are ‘frustrated with the exercises provided’ [36]. This highlights that patient preference could be critical when developing and refining strengthening programs. Accordingly, it would be worthwhile if future research investigated patient preferences in exercise prescription for PHP.

## Conclusion

In this study we used a Delphi technique to develop three progressive strengthening programs for three patient types (younger athletic adult, overweight middle-aged adult, and an older adult) who typically experience PHP. After three rounds, we found that all programs met consensus. The three programs can be used in future clinical trials to evaluate their effectiveness for PHP. In addition, clinicians could use the proposed programs with the caveat that they may change as future research findings become available.

### Supplementary Information

**Additional. file 1.** Patient vignettes

**Additional file 2.** Indications and contraindications (total represents the number of participants reporting each issue)

**Additional file 3.** Exercise selection (total represents the number of participants reporting each exercise)

**Additional file 4.** Muscles to be targeted (total represents the number of participants reporting each muscle or muscle group)

## Data Availability

The data analysed during this study are available from the corresponding author upon reasonable request.

## Abbreviations

ACSM: American College of Sports Medicine
PHP: Plantar heel pain
RM: Repetition maximum
