# Supplementary material for: Development of a foot and ankle strengthening program for the treatment of plantar heel pain: a Delphi consensus study
Source: J Foot Ankle Res. 2023 Oct 3;16:67. doi: 10.1186/s13047-023-00668-2 (PMC10546707; doi:10.1186/s13047-023-00668-2)
Supplement: Supplementary file 1 — Additional. file 1. Patient vignettes [file 13047_2023_668_MOESM1_ESM.docx]

**Additional file 1.** **Patient vignettes**

|  | **Younger athletic adult (Monique)** | **Overweight middle-aged adult (James)** | **Older adult**  **(Martha)** |
| --- | --- | --- | --- |
| Demographics/patient characteristics | | | |
| Sex | Female | Male | Female |
| Age | 25 years | 45 years | 75 years |
| Height | 160 cm (5 foot 3 inches) | 189 cm (6 feet 2 inches) | 165 cm (5 feet 4 inches) |
| Weight | 52 kg (114 lbs) | 121 kg (266 lbs) | 75 kg (165 lbs) |
| BMI | 20 kg/m2 | 33.9 kg/m2 | 27.5 kg/m2 |
| Background | | | |
|  | Over the past 6 months, Monique has developed right plantar heel pain that has been diagnosed as plantar fasciitis. She is training for her first full marathon (in 4 months) and is currently running 30 – 45 km/week (19 – 30 miles/week). She has run 3 half marathons in the past and has been running consistently for 6 years. Monique would like to reduce her pain and improve her function, and she has a goal of completing the full marathon. | Over the past 6 months, James has developed right plantar heel pain that has been diagnosed as plantar fasciitis. James’ weight has increased, and upon advice from his GP, he increased the frequency of his walking to reduce his weight. James would like to reduce his pain so he can continue walking to reduce his weight. | Over the past 6 months, Martha has developed right plantar heel pain that has been diagnosed as plantar fasciitis. She is very active and now finds the pain is limiting her ability remain active, which is starting to affect her mood. Martha would like to reduce her pain so she can continue her active lifestyle. |
| Social history | | | |
|  | Monique works as a manager for utilities company, mostly while seated at a desk. She lives at home with her partner, and running is her only form of exercise. | James is the General Manager of a real estate business. He is married with a family of 2 children, who are both early teenagers. | Martha lives on her own, however she maintains regular social contact with friends and family. She attends an ‘Active Seniors’ program 3 times per week at her local gym that involves various low-impact exercises classes, and she tries to walk every day for 4 km (2.5 miles) with her neighbour. |
| Medical history | | | |
|  | None reported | Hypertension | Hypertension |
| Medication history | Nil medication | 10 mg perindopril, orally, daily (an ACE inhibitor to manage hypertension) | 10 mg perindopril, orally, daily (an ACE inhibitor to manage hypertension) |
| Treatment history | Monique is currently using over-the-counter insoles which were purchased 1 month ago. She has been rolling her foot on an iced water bottle, and performing plantar fascia stretches for 1 month. | James is currently using over-the-counter insoles which were purchased 1 month ago. He has been rolling his foot on an iced water bottle, and performing plantar fascia stretches for 1 month. | Martha is currently using over-the-counter insoles which were purchased 1 month ago. She has been rolling her foot on an iced water bottle, and performing plantar fascia stretches for 1 month. |
| Assessment | | | |
| Symptoms | Symptoms occur with the first steps in the morning and upon rising after a long day on her feet. | Symptoms occur with the first steps in the morning and upon rising after a long day on his feet. | Symptoms occur with the first steps in the morning and upon rising after a long day on her feet. |
| Pain | Pain is sharp, and rated as 7/10 in the morning and 6/10 at the end of the day. | Pain is sharp, and rated as 7/10 in the morning and 6/10 at the end of the day. | 7/10 in the morning and 6/10 at the end of the day. |
| Palpation | Tender on palpation of medial calcaneal tubercle of right foot. | Tender on palpation of medial calcaneal tubercle of right foot. | Tender on palpation of medial calcaneal tubercle of right foot. |
| Single leg heel raise | 15 repetitions on each leg before fatigue. | 10 repetitions on each leg before fatigue. | 4 repetitions on each leg before fatigue. |
| Single leg balance | 15 seconds without pain. | 7 seconds without pain. | 7 seconds without pain. |
| Knee to wall | 12 cm (4.7 inches) bilaterally. | 7 cm (2.7 inches) bilaterally. | 4 cm (1.6 inches) bilaterally. |
| Diagnostic ultrasound | Plantar fascial thickening of 6 mm on the right foot and 3.9 mm on the left foot. | Plantar fascial thickening of 6 mm on the right foot and 3.9 mm on the left foot. | Plantar fascial thickening of 6 mm on the right foot and 3.9 mm on the left foot. |
