# Supplementary material for: Development of a foot and ankle strengthening program for the treatment of plantar heel pain: a Delphi consensus study
Source: J Foot Ankle Res. 2023 Oct 3;16:67. doi: 10.1186/s13047-023-00668-2 (PMC10546707; doi:10.1186/s13047-023-00668-2)
Supplement: Supplementary file 2 — Additional file 2. Indications and contraindications (total represents the number of participants reporting each issue) [file 13047_2023_668_MOESM2_ESM.docx]

**Additional file 2.** **Indications and contraindications (total represents the number of participants reporting each issue)**

| **Indication or contraindication** | **Total** |
| --- | --- |
| No restrictions | 6 |
| Physically active | 4 |
| Other pathology present | 3 |
| Measured weakness | 3 |
| Increased pronation | 2 |
| Chronic presentation | 2 |
| Pain on propulsion | 1 |
| Overly supportive shoes | 1 |
| BMI < 30 kg/m^2^ | 1 |
| Increased joint mobility | 1 |
| Failure to respond to other treatment | 1 |
