# Supplementary material for: Development of a foot and ankle strengthening program for the treatment of plantar heel pain: a Delphi consensus study
Source: J Foot Ankle Res. 2023 Oct 3;16:67. doi: 10.1186/s13047-023-00668-2 (PMC10546707; doi:10.1186/s13047-023-00668-2)
Supplement: Supplementary file 3 — Additional file 3. Exercise selection (total represents the number of participants reporting each exercise) [file 13047_2023_668_MOESM3_ESM.docx]

**Additional file 3. Exercise selection (total represents the number of participants reporting each exercise)**

| **Exercise** | **Variation** | **Total** |
| --- | --- | --- |
| Heel raises |  | 10 |
|  | Digits extended (dorsiflexed) | 4 |
|  | Concentric | 3 |
|  | Eccentric | 2 |
|  | Single leg | 1 |
| Digital plantarflexion |  | 8 |
| Short foot exercise |  | 8 |
| Digital extension |  | 3 |
| Digital adduction |  | 2 |
| Digital abduction |  | 2 |
| Toe spread out |  | 2 |
| Single leg standing |  | 2 |
| Ankle eversion |  | 1 |
| Ankle inversion |  | 1 |
| Hopping |  | 1 |
| Stretching |  | 2 |
