# Supplementary material for: Development of a foot and ankle strengthening program for the treatment of plantar heel pain: a Delphi consensus study
Source: J Foot Ankle Res. 2023 Oct 3;16:67. doi: 10.1186/s13047-023-00668-2 (PMC10546707; doi:10.1186/s13047-023-00668-2)
Supplement: Supplementary file 4 — Additional file 4. Muscles to be targeted (total represents the number of participants reporting each muscle or muscle group) [file 13047_2023_668_MOESM4_ESM.docx]

**Additional file 4.** **Muscles to be targeted (total represents the number of participants reporting each muscle or muscle group)**

| **Muscles** |  | **Total** |
| --- | --- | --- |
| Foot intrinsics |  | 6 |
| Flexor hallucis longus |  | 2 |
| Calf |  | 2 |
| Adductors |  | 1 |
| Flexor digitorum brevis |  | 1 |
| Tibialis posterior |  | 1 |
| Flexor digitorum longus |  | 1 |
